# A Comprehensive Review of Genetic Variations in Collagen-Encoding Genes and Their Implications in Intervertebral Disc Degeneration

**DOI:** 10.7759/cureus.52708

**Published:** 2024-01-22

**Authors:** Sachin Goel, Sanjay Deshpande, Nareshkumar Dhaniwala, Rahul Singh, Anmol Suneja, Vivek H Jadawala

**Affiliations:** 1 Orthopaedics, Jawaharlal Nehru Medical College, Datta Meghe Institute of Higher Education and Research, Wardha, IND

**Keywords:** genome-wide association studies, personalized medicine, molecular mechanisms, genetic variations, collagen-encoding genes, intervertebral disc degeneration

## Abstract

This comprehensive review examines the intricate relationship between genetic variations in collagen-encoding genes and their implications in intervertebral disc degeneration (IVDD). Intervertebral disc degeneration is a prevalent spinal condition characterized by structural and functional changes in intervertebral discs (IVDs), and understanding its genetic underpinnings is crucial for advancing diagnostic and therapeutic strategies. The review begins by exploring the background and importance of collagen in IVDs, emphasizing its role in providing structural integrity. It then delves into the significance of genetic variations within collagen-encoding genes, categorizing and discussing their potential impact on disc health. The methods employed in studying these variations, such as genome-wide association studies (GWASs) and next-generation sequencing (NGS), are also reviewed. The subsequent sections analyze existing literature to establish associations between genetic variations and IVDD, unraveling molecular mechanisms linking genetic factors to disc degeneration. The review concludes with a summary of key findings, implications for future research and clinical practice, and a reflection on the importance of understanding genetic variations in collagen-encoding genes to diagnose and treat IVDD. The insights gleaned from this review contribute to our understanding of IVDD and hold promise for the development of personalized interventions based on individual genetic profiles.

## Introduction and background

Intervertebral disc degeneration (IVDD) is a prevalent and debilitating condition affecting the spine, characterized by structural and functional changes in the intervertebral discs (IVDs). These discs provide flexibility, stability, and shock absorption to the spine. Intervertebral disc degeneration is associated with various factors, including aging, mechanical stress, and genetic predisposition [1]. Collagen, a fibrous protein, constitutes a significant component of the extracellular matrix (ECM) within IVDs. It provides tensile strength, resilience, and structural integrity to the discs. The unique composition of collagen fibers contributes to the ability of IVDs to withstand compressive forces and maintain their structural stability. Any alterations in collagen homeostasis can have profound implications for disc health [2].

Genetic variations within the genes encoding collagen, the primary structural protein of IVDs, have been increasingly recognized as potential contributors to the susceptibility and progression of IVDD. These variations may influence collagen synthesis, assembly, and degradation, thereby impacting the overall integrity of the IVD ECM. Understanding the genetic basis of IVDD is crucial for unraveling the complex etiology of this condition and developing targeted interventions [3]. This comprehensive review aims to critically examine the existing literature on genetic variations in collagen-encoding genes and their implications in IVDD. By synthesizing current knowledge, we aim to shed light on the molecular mechanisms through which genetic variations affect collagen function, ultimately contributing to IVDD. Additionally, this review will explore the potential clinical applications, challenges, and future directions for harnessing genetic insights for diagnosing, prognosing, and treating IVDD.

## Review

Collagen structure and function

Overview of Collagen

Collagen stands as the most abundant protein in the animal kingdom, serving as a foundational support in the extracellular space of connective tissues. This fibrous protein adopts a right-handed bundle structure of three parallel, left-handed polyproline II-type helices. Within the human body, collagen holds a significant presence, constituting one-third of the total protein and contributing to three-quarters of the dry weight of the skin. Its distribution extends to connective tissue, tendons, ligaments, cartilage, bones, and skin, making up 30% of the body's protein composition [4-6].

The structural hallmark of collagen lies in its triple-helical domain, which comprises three distinct alpha chains. Synthesis initiates within specialized fibroblast cells, where amino acids undergo activation and peptide subunits assemble on the ribosome. Collagen manifests in various types, from type I to type V, each fulfilling distinct functions. Discrepancies in collagen synthesis can result in diverse clinical manifestations, exemplified in conditions such as scurvy, osteogenesis imperfecta, and Ehlers-Danlos syndrome [5, 6].

The mechanical and structural attributes of native collagen are pivotal to its functionality, providing essential tensile strength. As a vital extracellular matrix component, collagen plays a critical role in shaping fibrillar and microfibrillar networks across diverse tissues [4,5]. Its unique triple-helical structure bestows strength and stability, establishing collagen as a critical structural protein with widespread functions. Given its abundance and diverse roles, collagen is crucial for maintaining the integrity of various tissues and organs within the human body.

Collagen Types and Distribution in Intervertebral Discs

The intervertebral disc is a highly specialized cartilaginous tissue showcasing various collagens, including types I, II, III, and VI. Notably, type II collagen is the most abundant, constituting 50%-70% of the total collagen content, while type I collagen predominantly resides in the outer annulus fibrosus. Type III collagen, isolated from average human and bovine intervertebral discs, is primarily localized around cells. Additionally, type VI collagen is found within the intervertebral disc, exhibiting unusual abundance among the minor collagens in bovine disc tissue. The collagen framework of the intervertebral disc is characterized by the presence of two major fibril-forming collagens, types I and II, complemented by smaller amounts of other collagen types [7-9].

The distribution of distinct molecular collagen species within the intervertebral disc varies, with type I exclusively at the extreme outer edge and type II exclusive to the nucleus pulposus [10]. The diverse types of collagen in the intervertebral disc significantly contribute to its mechanical and structural properties, imparting tensile strength. This intricate collagen composition plays a pivotal role in establishing fibrillar and microfibrillar networks across various tissues, ultimately shaping the mechanical integrity of the intervertebral disc [7].

Role of Collagen in Maintaining Disc Structure and Function

Collagen plays a pivotal role in upholding the structural integrity and functionality of the IVD, a complex structure comprised of the annulus fibrosus (AF) and the nucleus pulposus (NP). These components house different types of collagen, contributing distinct properties to the overall function of the disc. The AF, encircling the NP in a ring-shaped structure, is primarily composed of 15 to 25 stacked sheets rich in type I collagen. This collagen type imparts tensile strength to the AF, enabling the disc to endure compressive forces. Additionally, the collagen framework of the IVD includes an unusually abundant presence of type VI collagen among the minor collagens within the disc tissue [9,11].

Conversely, the NP, located at the disc's center, adopts a gel-like structure with a water content ranging from 66% to 86%. Predominantly composed of type II collagen and types VI, IX, and XI, the NP relies on this collagen type to maintain its gel-like properties and retain water. Type II collagen is integral to the IVD's capacity to absorb and distribute compressive loads, providing essential resistance to tensile forces [11,12].

Studies mapping collagen content and structure in human IVD degeneration reveal alterations indicative of fibrosis, with a shift from collagen type II to type I within the disc. While IVD degeneration is often associated with a fibrotic process, conclusive evidence of increased collagen content reflective of fibrosis remains elusive [13]. The diversity of collagen types within the IVD is indispensable for preserving its structural integrity and functionality. Type I collagen in the AF contributes to tensile strength, whereas type II collagen in the NP enhances the disc's resilience against compressive forces and aids in water retention. The harmonious balance and distribution of these collagens are paramount for the mechanical properties of the IVD and its ability to resist degeneration.

Genetic basis of collagen variations

Introduction to Genetic Variations

Genetic variations within collagen-encoding genes play a pivotal role in shaping human health, considering the crucial role of collagens as essential structural proteins. Mutations in these genes can give rise to a spectrum of disorders with significant clinical implications. A comprehensive study investigating natural variation in four human collagen genes across diverse populations identified many single nucleotide polymorphisms (SNPs), 15 resulting in amino acid substitutions [14]. Notably, mutations in the COL1A1 and COL1A2 genes, responsible for encoding type I collagen, have been linked to the weakening of connective tissues and the onset of conditions such as osteogenesis imperfecta and Ehlers-Danlos syndrome [15,16]. Similarly, gene mutations encoding type IV collagens have been associated with various health conditions, particularly those affecting the kidneys [17]. Recognizing and comprehending the genetic basis of collagen variations is paramount for unraveling the etiology of related disorders and, crucially, for developing targeted treatments to address the underlying genetic factors contributing to these health challenges.

Types of Genetic Variations in Collagen-Encoding Genes

Single nucleotide polymorphisms: These represent a prevalent form of genetic variation in collagen-encoding genes, involving a single nucleotide alteration in the DNA sequence. A comprehensive study examining natural variations in four human collagen genes across diverse populations identified 459 SNPs, 15 of which resulted in amino acid substitutions [14]. Notably, SNPs within collagen-encoding genes have been linked to various health conditions, including exercise-induced muscle damage, osteoporosis, and anterior cruciate ligament rupture [18, 19]. Grasping the role of SNPs in collagen genes is imperative for understanding the etiology of associated disorders and developing targeted treatment strategies.

Insertions and deletions (indels): Genetic variations in collagen-encoding genes encompass diverse mutations, such as SNPs, insertions, and deletions (indels). Mutations within genes encoding type I collagen, like COL1A1 and COL1A2, can result in conditions such as osteogenesis imperfecta and Ehlers-Danlos syndrome [14, 15]. Similarly, mutations in genes encoding type IV collagens have been correlated with kidney disorders and other health conditions [16]. A thorough investigation of natural variation in four human collagen genes across diverse populations identified 459 SNPs, with 15 leading to amino acid substitutions [14]. These mutations impact the physiochemical properties of both native and altered collagens, contributing to weakened connective tissue and associated health conditions [15]. A nuanced understanding of the various genetic variations in collagen-encoding genes is essential for comprehending the etiology of related disorders and developing targeted treatments.

Copy number variations (CNVs): Genetic variations within collagen-encoding genes manifest in various forms, including SNPs, CNVs, and mutations. Copy number variations, constituting structural alterations in the genome involving the deletion or duplication of DNA segments, play a significant role. An extensive analysis of natural variation in four human collagen genes across diverse populations identified 459 SNPs, with 15 resulting in amino acid substitutions [14]. Mutations within the COL1A1 and COL1A2 genes, responsible for encoding type I collagen, can weaken connective tissue, leading to conditions such as osteogenesis imperfecta and Ehlers-Danlos syndrome [15, 20]. Furthermore, CNVs in collagen-encoding genes have been associated with diverse health conditions, including osteoporosis and osteoarthritis [21]. An in-depth understanding of the diverse genetic variations within collagen-encoding genes is crucial for comprehending the etiology of related disorders and developing targeted treatment approaches.

Identification and Classification of Collagen-Encoding Genes

COL1A1 and COL1A2: These genes play a pivotal role in encoding the chains of type I collagen, the most abundant collagen in the human body. Type I collagen is a fundamental component of bones, skin, tendons, and other connective tissues. Mutations in COL1A1 and COL1A2 have profound implications, leading to osteogenesis imperfecta, a debilitating condition characterized by brittle bones and weakened connective tissues [14, 15]. The impact of these mutations extends beyond skeletal integrity, emphasizing the crucial role of type I collagen in maintaining the structural integrity of various tissues throughout the body.

COL2A1: The COL2A1 gene encodes the chains of type II collagen, primarily found in cartilage and intervertebral discs. Type II collagen is crucial for maintaining these tissues' structural integrity and resilience. Mutations in COL2A1 can result in chondrodysplasias, a group of disorders characterized by abnormal growth of bones and cartilage [14]. This highlights the critical role of type II collagen in ensuring proper skeletal development and the consequences when its genetic blueprint is altered.

COL3A1: Encoding the chains of type III collagen, found in diverse tissues such as skin, tendons, and blood vessels, the COL3A1 gene is vital for maintaining tissue strength and elasticity. Mutations in COL3A1 can lead to vascular Ehlers-Danlos syndrome, a condition characterized by weakened connective tissue and various health complications [14]. The diverse tissue distribution of type III collagen underscores its significance in ensuring the structural integrity of multiple bodily systems.

COL9A1, COL9A2, and COL9A3: These genes collectively encode the chains of type IX collagen, a key component found in cartilage and corneas. COL9A1, COL9A2, and COL9A3 mutations have been linked to a specific collagen disorder known as type IX [14]. Understanding the genetic basis of this disorder is crucial for unraveling the intricate role of type IX collagen in maintaining the structural stability of cartilage and corneal tissues.

COL11A1 and COL11A2: Responsible for encoding the chains of type XI collagen distributed in tissues such as skin and ligaments, COL11A1 and COL11A2 are crucial for structural support and elasticity. Mutations in these genes have been associated with type XI collagen disorder [20]. This underscores the importance of type XI collagen in maintaining the integrity of various tissues beyond skeletal elements, emphasizing its role in skin and ligament function.

Methods for studying genetic variations

Genome-Wide Association Studies (GWAS)

Genome-wide association studies are potent for discerning genetic variations linked to specific traits or diseases. The execution of a GWAS involves several pivotal steps, encompassing quality control for both samples and variants, rigorous statistical analysis, and the subsequent deposition of data [22]. Various methods and tools, such as Human Splicing Finder, MaxEntScan, NNSplice, and SplicePort, are utilized to evaluate the impact of genetic variations [23]. In the context of endangered species, the assessment of genetic variation commonly relies on genetic markers like allozymes, microsatellites, and SNPs [24]. Moreover, the study of genetic variation encompasses diverse approaches, including genome-wide association studies, investigations into the functional consequences of variants, and population genetics [25]. These methodologies and tools are indispensable for comprehending the intricate role of genetic variations across a spectrum of traits and diseases, offering valuable insights into their implications for human health and evolution.

Next-Generation Sequencing (NGS)

Next-generation sequencing (NGS) has brought about a revolutionary transformation in investigating genetic variations, facilitating a thorough examination of the genome. Next-generation sequencing allows for sequencing the complete genome and specific genes through gene panels or protein-coding regions via exome sequencing. This is achieved at a notably reduced cost and with heightened throughput compared to conventional methods like Sanger sequencing [26]. Next-generation sequencing-based techniques utilized for scrutinizing genetic variations and their correlation with specific phenotypes encompass genome or exome sequencing and gene panels. These methodologies have played a substantial role in uncovering rare mutations linked to complex phenotypes, which pose significant challenges with traditional sequencing techniques [27, 28].

In evaluating the impact of genetic variations, diverse approaches, and tools have been devised, including Human Splicing Finder, MaxEntScan, NNSplice, and SplicePort. These tools prove indispensable for predicting the functional repercussions of genetic variations and find extensive use among researchers and clinicians [23]. Targeted NGS also serves as a precision medicine tool, allowing for the swift identification of common and rare genetic variations. This approach is precious for pinpointing variants contributing to therapeutic drug responses or adverse effects and involves using quality-based variant detection tools [29]. Next-generation sequencing has significantly propelled the exploration of genetic variations by enabling comprehensive genome analysis, the identification of rare mutations, and the development of tools for evaluating the impact of genetic variations. These methodologies have markedly enhanced our comprehension of the genetic underpinnings of various phenotypes, with far-reaching implications for both research endeavors and clinical applications.

Bioinformatic Tools for Collagen Gene Analysis

Bioinformatic tools play a crucial role in examining genetic variations within collagen-encoding genes. Next-generation sequencing tools, such as whole exome sequencing (WES) or customized target panels, offer the capability to scrutinize specific regions or the entire exome, aiding in the identification of genes responsible for diseases [30]. While Sanger sequencing has traditionally served as the gold standard for genetic confirmation, its cost and efficiency limitations make it less suitable for the heterogeneous nature of next-generation sequencing [30]. Bioinformatics tools also prove valuable in classifying collagen from diverse animal sources based on physicochemical properties and other analyzed features [31].

Several approaches and tools have been devised to assess the impact of genetic variations, including Human Splicing Finder, MaxEntScan, NNSplice, and SplicePort [23]. These tools play a pivotal role in predicting the phenotypic consequences of genetic variations. Extensive research has been conducted on genetic variations in collagen-encoding genes in the specific context of intervertebral disc degeneration (IVDD). Notably, mutations in the COL9 gene have been identified as influencing disc degeneration in mice and humans [32]. Furthermore, the rs1800012 polymorphism of the COL1A1 gene has been linked to an increased risk of IVDD [33]. A comprehensive understanding of the etiology behind impaired collagen formation holds the potential to guide medical professionals in prescribing the most effective treatments for IVDD [33].

Association of genetic variations with intervertebral disc degeneration

Review of Relevant Studies

Numerous studies have investigated the intricate relationship between genetic variations and IVDD. Variants within genes crucial for developing and maintaining intervertebral discs and vertebrae have been identified as associated with IVDD [32]. Notably, the genes most frequently linked to IVDD provide instructions for synthesizing collagen proteins [34, 35]. Variations in these structural genes may render intervertebral discs more susceptible to mechanical loading, potentially resulting in injuries to the endplates and annulus fibrosus, ultimately leading to a loss of disc function [36]. Furthermore, genetic variants in genes encoding cytokines, such as interleukin-1 (IL1) and IL6, as well as degradative metalloproteinases (MMPs), have been reported to be associated with IVDD [35, 36]. Recent comprehensive studies, including genome-wide association studies, have uncovered novel variants within various genes contributing to the risk of intervertebral disc degeneration and subsequent back pain [35]. The heritability of IVDD has been established through twin studies, with familial aggregation explaining a substantial proportion of lumbar disc degeneration [35]. The significance of genetic variations in the development of IVDD is evident, and further research is imperative to comprehensively understand the mechanisms through which these genetic alterations contribute to IVDD development and to identify potential therapeutic targets [34, 35].

Correlation Between Specific Genetic Variations and IVDD Risk

Genetic variations have emerged as significant contributors to IVDD, with studies identifying specific genetic variations correlating with the risk of developing this condition. Notably, variations in the IL6 gene have been associated with IVDD characterized by sciatica, shedding light on the potential role of inflammatory pathways in the pathogenesis of IVDD [35]. Moreover, polymorphisms in genes encoding collagens, including COL1A1, COL9A1, COL9A2, COL9A3, COL11A1, and COL11A2, have been identified as critical factors influencing susceptibility to IVDD [33, 35]. These findings underscore the intricate involvement of genetic factors in the development of IVDD.

The heritability of IVDD has been firmly established through twin studies, where genetic variation has been recognized as a discernible risk factor for the condition [35]. Notably, the focus of research has been on the structural components of the intervertebral disc, particularly genes encoding collagens, due to their potential impact on disc vulnerability to mechanical loading and subsequent loss of function [36]. While the precise mechanisms underpinning the correlation between specific genetic variations and the risk of IVDD remain incompletely understood, the identified associations provide valuable insights into the genetic basis of this common spinal pathology.

Understanding the role of genetic variations in IVDD holds promise for developing more effective diagnostic and therapeutic approaches. Insights into the genetic landscape of IVDD enhance our comprehension of its etiology and open avenues for personalized interventions, ultimately contributing to improved clinical management of this prevalent spinal disorder [33].

Gene-Environment Interactions

Genetic variations exert a significant influence on IVDD, a leading cause of low back pain with a complex etiology involving both genetic and environmental factors. Various genes, including those encoding collagens I, II, III, IX, and XI, proteoglycans, cytokines, and enzymes, have been implicated in IVDD. These genetic factors contribute to a broad spectrum of variations, such as SNPs, that play a substantial role in disease development [37]. Current research suggests that genetic factors account for approximately 75% of susceptibility to IVDD, with identified polymorphisms in genes associated with type I, IX, and XI collagens potentially influencing disease progression [37]. Specific genetic variations have also been linked to an increased risk of IVDD, underscoring the importance of genetic predisposition in this condition [34, 38]. While the mechanisms through which genetic variations contribute to IVDD are not fully understood, it is hypothesized that variations in structural genes may render intervertebral discs more susceptible to mechanical loading, leading to the loss of disc function and the onset of IVDD [36]. Genetic variations, including single nucleotide polymorphisms in various genes, play a significant role in the etiology of intervertebral disc degeneration. Further research is crucial to unraveling the specific genetic mechanisms and their interactions with environmental factors in the development of IVDD. Continued exploration of these intricate relationships holds the promise of advancing our understanding and facilitating the development of more targeted approaches for diagnosing and treating this prevalent spinal pathology.

Molecular mechanisms underlying the impact of genetic variations

Altered Collagen Expression and Synthesis

Regulating collagen expression and synthesis through genetic variations involves a complex array of molecular mechanisms. Control over collagen gene expression is exerted through transcriptional, posttranscriptional, and translational mechanisms, which respond to biological and pharmacological inducers [39]. Notably, mutations in the COL1A1 and COL1A2 genes, responsible for encoding type I collagen, are linked to rare genetic disorders like osteogenesis imperfecta and specific types of Ehlers-Danlos syndrome [40]. These mutations can result in modified collagen synthesis, leading to structural abnormalities that impact collagen's thermal stability and overall function [40]. Additionally, mutations in the COL9 gene, responsible for encoding collagen IX, have demonstrated an association with intervertebral disc degeneration in mice and humans [32]. The diverse molecular mechanisms influenced by genetic variations in collagen-encoding genes can give rise to significant clinical manifestations, contributing to various genetic disorders and conditions, including intervertebral disc degeneration.

Effects on Extracellular Matrix Integrity

Maintaining tissue structure and function critically relies on the integrity of the ECM. Genetic variations, particularly those impacting collagen expression and synthesis, influence ECM integrity. Current research suggests that genetic variations leading to altered collagen expression can result in the degradation and remodeling of the ECM, consequently affecting the function of surrounding cells and tissues [41]. Notably, mutations in collagen-encoding genes have been linked to conditions such as osteogenesis imperfecta and intervertebral disc degeneration, underscoring the intricate connection between genetic variations, collagen, and ECM integrity [42, 43].

Moreover, a study has highlighted the essential role of mitochondrial respiratory chain function in promoting ECM integrity in cartilage, emphasizing the contribution of cellular mechanisms to maintaining ECM stability [42]. Additionally, the structural integrity of the ECM has been shown to influence the mechanical behavior of chondrocytes, indicating a nuanced relationship between ECM integrity and cellular function [44]. Genetic variations, especially those influencing collagen expression, affect ECM integrity, impacting tissue structure and function. A comprehensive understanding of the molecular mechanisms governing these effects is crucial for elucidating the pathogenesis of various conditions and holds the potential for developing therapeutic interventions.

Influence on Cellular Processes in Intervertebral Discs

Genetic variations, particularly those affecting genes responsible for encoding collagen, substantially influence cellular processes within intervertebral discs, playing a crucial role in developing IVDD, a prevalent cause of low back pain. Extensive research indicates that genetic defects correlated with structural and functional alterations in the intervertebral disc can compromise its mechanical properties and metabolic activities, thereby contributing to the onset and progression of IVDD [32].

Specifically, mutations in genes encoding collagens, including COL9 and COL11, have been identified as critical factors associated with disc degeneration in mice and humans, underscoring the genetic underpinnings of IVDD [32, 35]. These genetic variations have the potential to impact the mechanical properties of disc tissue by causing a decrease in synthesis and an increase in the breakdown of the extracellular matrix, crucial factors in the development of IVDD [32]. While the precise mechanisms through which genetic variations influence cellular processes in intervertebral discs remain incompletely understood, it is evident that genetic factors play a significant role in the etiology of IVDD. Further research in this area is imperative to enhance our comprehension of the molecular basis of IVDD, paving the way for developing potential therapeutic interventions.

Clinical implications

Genetic Testing for IVDD Risk Prediction

The prospect of genetic testing for predicting the risk of intervertebral disc degeneration (IVDD) has garnered attention, driven by the recognition of the condition's complex etiology involving genetic and environmental factors. Numerous studies have reported associations between polymorphisms in genes responsible for encoding collagen and susceptibility to IVDD. Notably, the rs1800012 polymorphism in the COL1A1 gene has been linked to an elevated risk of IVDD [33, 45]. Moreover, research has revealed that genetically predicted triglycerides (TG) play a mediating role in the association between type 2 diabetes mellitus (T2DM) and IVDD, with TG accounting for a substantial portion of the heightened IVDD risk [46]. While the role of genetic testing in predicting IVDD risk is still evolving, the identification of genetic factors, particularly in collagen-encoding genes, presents an avenue for personalized risk assessment and targeted interventions. Nevertheless, the full clinical implications of genetic testing for IVDD risk prediction necessitate further research and validation in large clinical cohorts to establish its reliability and utility in a broader clinical context.

Personalized Medicine Approaches

In the continually evolving healthcare landscape, personalized medicine, also known as precision medicine, stands out as a transformative paradigm that considers individual variations in genes, environment, and lifestyle when devising medical interventions [47,48]. This approach harnesses genetic information to tailor healthcare strategies, spanning disease prevention, diagnosis, and treatment. At its core, personalized medicine recognizes the distinctive physiological, environmental, and behavioral profiles of individuals, advocating for interventions that are finely tuned to their specific health conditions [47, 48]. The clinical implications of personalized medicine are substantial, offering the potential to shift medicine from a reactive to a preventive paradigm. This paradigm empowers healthcare providers to predict disease susceptibility, enhance disease detection, intervene preemptively in disease progression, and customize disease-prevention strategies. Moreover, it enables healthcare professionals to prescribe more effective drugs, circumvent medications with predictable side effects, and optimize resource utilization by avoiding ineffective treatments [48]. However, despite its promise, implementing personalized medicine is not without challenges. Paramount among these challenges is the necessity of establishing the clinical utility of personalized medicine protocols. Ethical, legal, and social implications pose additional hurdles, as do issues related to cost, healthcare accessibility, and information technologies [49]. Challenges extend to interpreting complex model predictions and the imperative for validation through prospective clinical trials, which currently hinders seamless integration into routine clinical practice [50]. While personalized medicine holds immense potential for enhancing patient care by tailoring medical decisions to individual characteristics, its full realization necessitates addressing various challenges, including ethical considerations, cost constraints, and rigorous clinical validation. Overcoming these challenges is crucial for facilitating the broader adoption of personalized medicine and maximizing its impact on healthcare practices [49].

Challenges and Limitations in Translating Genetic Findings to Clinical Practice

Translating genetic findings into clinical practice is a formidable task, marked by various limitations. A primary challenge lies in the necessity of the widespread availability of sequence data and its correlation with clinical information to ensure the accuracy and validity of results [51]. Ethical considerations pose another hurdle, particularly concerning the return of all genetic information to patients, even when deemed clinically irrelevant by the laboratory and clinician [51]. Elaborating on the ethical considerations, it is essential to delve into specific examples of ethical dilemmas in genetic testing for IVDD. For instance, one ethical dilemma arises in the context of incidental findings, where genetic testing may reveal information unrelated to the primary reason for testing. This raises questions about whether and how such incidental findings should be communicated to patients, considering their potential psychological and medical implications.

Locus heterogeneity further complicates matters, impacting the accuracy of genetic testing by adding time and cost to the testing process, necessitating testing of an affected proband for precise interpretation [51]. Furthermore, the interpretation of genetic variants remains a significant challenge, with most identified variants holding unknown clinical significance due to factors such as incomplete penetrance and variable expressivity [52]. Deciding which variants should be included in a formal clinical report is critical, emphasizing the need to consult research-based evaluations for further clarification [52]. Moreover, the cost-effectiveness of genomic technologies varies across different subpopulations, with the inherent characteristics of genetic tests contributing to increased complexity in analyses [53]. This introduces ethical considerations related to healthcare disparities, as certain populations may face barriers to accessing genetic testing due to financial constraints or other systemic factors. Addressing these ethical challenges is crucial for ensuring equitable access to genetic information and preventing further disparities in healthcare. While incorporating genetic testing and counseling has become standard for several diseases, the interpretation of genetic variants and the cost-effectiveness of genomic technologies persist as major hurdles in integrating genetics into routine clinical care [52]. Ethical considerations add a layer of complexity, requiring thoughtful and transparent approaches to navigate dilemmas such as incidental findings, locus heterogeneity, and healthcare disparities. Addressing these challenges is imperative to harness the full potential of genetic information for improved patient outcomes and personalized healthcare.

## Conclusions

In conclusion, the review highlights the pivotal role of genetic variations in collagen-encoding genes in the complex landscape of IVDD. Summarizing the key findings, it becomes evident that these genetic variations significantly influence the susceptibility and progression of IVDD by impacting collagen synthesis, assembly, and degradation. The molecular insights gained from this exploration offer a deeper understanding of the pathogenesis of IVDD but also present potential avenues for targeted therapeutic interventions. Looking ahead, the implications for future research and clinical practice are substantial. The identification of specific genetic markers associated with IVDD opens doors to personalized medicine, allowing for early risk assessment and tailored treatment strategies. The discovered therapeutic targets arising from genetic insights call for further investigation, holding promise for developing novel interventions. However, translating these genetic discoveries into practical tools for clinical use requires ongoing collaborative efforts and the standardization of genetic testing methodologies. In closing, the review emphasizes the importance of understanding genetic variations in collagen-encoding genes in the context of IVDD, offering a comprehensive view of the condition and paving the way for a more precise and personalized approach to its diagnosis and treatment.
